# A Federally Qualified Health Center pilot project for early screening and outreach in underserved areas in southern Indiana

**DOI:** 10.1002/alz70860_100452

**Published:** 2025-12-23

**Authors:** Lori Harris

**Affiliations:** ^1^ Family Health Centers of Southern Indiana, Jeffersonville, IN, USA

## Abstract

**Background:**

Family Health Centers of Southern Indiana (FHCSI) is a group of Federally Qualified Health Centers (FQHC) serving a diverse, high‐risk population, with 87% of patients below 200% of the federal poverty level. The patient population faces significant social determinants of health including poverty, food insecurity, limited education, addiction, and language barriers. These factors, combined with limited access to preventive care, place patients at elevated risk for cognitive impairment at an early age. Additionally, 70% of patients are uninsured or underinsured, limiting access to traditional Medicare Annual Wellness Visits for cognitive screening.

**Method:**

As part of the Davos Alzheimer's Collaborative Healthcare System Preparedness U.S. Early Detection Fellowship Program, FHCSI will increase outreach and assessments for Alzheimer's disease and related dementias at an early stage to increase screening rates and referrals to early intervention resources among underserved populations. This program will implement two approaches: 1) outreach to community safety net agencies to promote brain health screening awareness and to establish patient referral resources and 2) create standardized Mini‐Cog screening for all primary care visits for patients age 50+ at four clinic locations.

**Result:**

As part of this program, FHCSI plans to screen ≥85% of the current patient population aged 50+ through clinical visits and community outreach events. Efforts will focus on reaching uninsured, undocumented or non‐English speaking populations who may not be aware of the importance of early detection or do not prioritize preventative brain health screenings over acute care. The FQHC's billing structure allows for comprehensive screening services regardless of insurance status and will help ensure program sustainability. Early learnings in program implementation will be shared at the time of presentation.

**Conclusion:**

Through the U.S. Fellowship Program, FHCSI can enhance earlier detection (age 50+) and intervention of brain health decline in underserved/vulnerable populations in a vital public health clinical setting. FHCSI is uniquely positioned as a safety‐net provider, combined with its established community presence and FQHC status, to implement early screening in a demographic facing multiple risk factors for cognitive impairment. The dual approach of clinical integration and community outreach will ensure comprehensive program reach while creating long‐term sustainability.

